# From inflammation to fibrosis and cancer: the emerging role of AIEC-derived metabolites in intestinal disease progression

**DOI:** 10.1038/s12276-026-01725-z

**Published:** 2026-05-19

**Authors:** Ju-Hyun Ahn, Anthony Hazelton, Juliane Nguyen, Janelle C. Arthur

**Affiliations:** 1 Department of Microbiology and Immunology, University of North Carolina at Chapel Hill, Chapel Hill, NC USA; 2 Division of Pharmacoengineering and Molecular Pharmaceutics, Eshelman School of Pharmacy, University of North Carolina at Chapel Hill, Chapel Hill, NC USA; 3 Lineberger Comprehensive Cancer Center, University of North Carolina at Chapel Hill, Chapel Hill, NC USA; 4 Center for Gastrointestinal Biology and Disease, University of North Carolina at Chapel Hill, Chapel Hill, NC USA

**Keywords:** Mechanisms of disease, Crohn's disease, Chronic inflammation, Infection, Colon cancer

## Abstract

The intestinal microbiota maintains mucosal homeostasis through dynamic host–microbe interactions. When this balance is disrupted, gut dysbiosis drives inappropriate immune activation, leading to dysregulated inflammation that contributes to the pathogenesis of chronic inflammatory diseases, including inflammatory bowel diseases (IBD). Chronic inflammation in patients with IBD increases the risk of intestinal fibrosis and colorectal cancer. However, therapeutic options for patients with IBD with fibrosis or neoplasia remain limited and challenging. Adherent-invasive *Escherichia coli* (AIEC) have emerged as key metabolic drivers of disease in IBD. We previously demonstrated that the AIEC-derived genotoxin colibactin and the siderophore yersiniabactin (Ybt) promote tumorigenesis through DNA damage and fibrosis via pro-fibrotic macrophage–fibroblast interactions, respectively. Because fibrosis and tumorigenesis involve overlapping pathways such as extracellular matrix remodeling, transforming growth factor-beta signaling, angiogenesis, and epithelial-to-mesenchymal transition, AIEC-derived metabolites may be functionally interconnected and could drive distinct pathological outcomes depending on the context of the disease. This Review highlights how AIEC-derived metabolites amplify inflammation, fibrosis, and neoplasia, outlines potential crosstalk between colibactin and Ybt, and discusses therapeutic opportunities targeting AIEC metabolite production in parallel with host-directed antifibrotic and cancer-prevention strategies.

## Colibactin and Ybt link inflammation to cancer risk

Chronic inflammation in the gut, particularly in conditions such as inflammatory bowel diseases, can lead to severe complications such as intestinal fibrosis and colorectal cancer. This Review explores the role of adherent-invasive *Escherichia coli* (AIEC), focusing on how AIEC-derived metabolites, such as colibactin and yersiniabactin (Ybt), contribute to disease progression. The authors highlight that AIEC persist in the inflamed gut by exploiting metabolic pathways and evading host defenses, driving sustained inflammation and tissue remodeling. Their research shows that colibactin induces DNA damage, promoting colorectal cancer, whereas Ybt disrupts metal homeostasis, contributing to fibrosis. More broadly, fibrosis and tumorigenesis share pathways such as extracellular matrix remodeling and transforming growth factor-beta signaling, suggesting that immune–stromal interactions warrant further investigation. Targeting AIEC-derived metabolites offers a potential therapeutic strategy for inflammation-associated intestinal diseases and cancer prevention.

This summary was initially drafted using artificial intelligence, then revised and fact-checked by the author.

## Introduction

Inflammation is essential for host defense against infection and has a critical role in tissue repair, regeneration, remodeling, and the maintenance of tissue homeostasis^1,2^. However, when inflammation becomes chronic, it alters the tissue microenvironment (TME) and contributes to the onset and progression of various diseases^2,3^.

The gut contains trillions of microorganisms, known as microbiota, that help maintain microbial and immune balance^4^. Disrupting this balance influences many human diseases, including inflammation and cancer. Inflammatory bowel diseases (IBDs), including Crohn’s disease (CD) and ulcerative colitis, are characterized by idiopathic, chronic inflammation of the gastrointestinal (GI) tract^3,5^. Despite extensive research, their etiology and pathogenesis remain incompletely understood. IBD presents diarrhea, abdominal pain, GI bleeding, and weight loss. Patients typically experience alternating periods of flare and remission, which substantially impair quality of life.

IBD arises from an abnormal immune response in genetically susceptible individuals, driven by complex interactions among host genetics, environmental factors, and the intestinal microbiota^5^. Normally, these factors maintain intestinal homeostasis; however, when dysregulated, they induce chronic immune-mediated inflammation and promote various comorbidities. This disruption, termed gut microbiome dysbiosis, is characterized by an enrichment of Proteobacteria and a depletion of beneficial commensals such as Firmicutes and Bacteroides, resulting in reduced microbial diversity^4^. Among the dysbiosis-associated bacteria, adherent-invasive *Escherichia coli* (AIEC) are strongly linked to ileal dysbiosis and CD pathogenesis^6–10^. Unlike classical enteric pathogens, AIEC lack traditional virulence factors but possess the ability to adhere to and invade intestinal epithelial cells (IECs) and to survive and replicate within macrophages^9–11^. However, the precise mechanisms connecting AIEC to disease progression remain incompletely defined. More recently, AIEC colonization has been implicated not only in inflammation but also in the development of intestinal fibrosis^12–16^. We have demonstrated that AIEC-produced yersiniabactin (Ybt) promotes intestinal fibrosis via immune–stromal cell activation^12,15^.

Chronic inflammation promotes DNA damage, oxidative stress, and aberrant activation of signaling pathways such as nuclear factor kappa-light-chain-enhancer of activated B cells (NF-κB) and signal transducer and activator of transcription 3 (STAT3), which together create a mutagenic microenvironment^2,3,17^. Patients with IBD exhibit an increased risk of colorectal cancer (CRC), with molecular pathways similar to those in sporadic cases — including mutations in adenomatous polyposis coli (*APC*) and tumor protein 53 (*TP53*) genes and activation of oncogenic Kirsten Rat Sarcoma Viral Oncogene Homolog (*KRAS*) and Cyclooxygenase 2 (*COX-2*) genes — but the sequence and inflammatory context in which these pathways are activated differ^2,3,17^. Consequently, managing neoplasia in patients with IBD, or treating IBD in those with a prior history of malignancy, remains a major clinical challenge owing to the limited understanding of underlying mechanisms. Dysbiosis further contributes to tumorigenesis, as CRC stool and tumor tissues often show enrichment of pro-inflammatory species such as *Fusobacterium nucleatum*, enterotoxigenic *Bacteroides fragilis*, and *E. coli*, alongside depletion of beneficial commensals such as Lachnospiraceae^18–22^. Furthermore, certain *E. coli* strains (*pks*^+^ *E. coli*) that produce colibactin, a small-molecule genotoxin, can directly induce DNA damage and promote colorectal carcinogenesis^23,24^. These findings underscore that certain microbes within the dysbiotic microbiota can actively drive tumorigenic processes via metabolite production and host–microbe interactions.

Importantly, IBD comorbidities such as fibrosis and tumorigenesis share overlapping molecular pathways, including extracellular matrix (ECM) remodeling, transforming growth factor-beta (TGF-β) signaling, angiogenesis, and epithelial-to-mesenchymal transition (EMT), which are often influenced by microbial metabolites and chronic inflammatory cues. Therefore, AIEC-derived metabolites may functionally bridge these processes, amplifying pathological remodeling and neoplastic transformation in a context-dependent manner. This Review highlights how AIEC-derived metabolites amplify inflammation, fibrosis, and neoplasia, examines potential crosstalk between colibactin and Ybt, and discusses therapeutic strategies targeting their role in the host. Furthermore, it explores emerging interventions such as engineered probiotic bacteria and yeast, host-directed anti-fibrotic and cancer-preventive approaches, emphasizing potential biomarkers for therapeutic response.

## AIEC and chronic inflammation

AIEC are abnormally associated with the ileal mucosa of patients with CD^6–10^. Although AIEC lack the classical virulence factors typical of enteric pathogens, they harbor multiple virulence-associated genes that enable adhesion to and invasion of IECs and survival within macrophages^9–11^. Since their initial discovery, AIEC pathogenic mechanisms have been well characterized in preclinical models; however, the genetic basis underlying AIEC–host interactions in patients with CD remains incompletely understood^9,25^. The absence of specific molecular markers distinguishing AIEC, coupled with heterogeneous pathoadaptive mutations and variable gene expression patterns, complicates precise strain identification^26,27^. Moreover, these genetic features are neither conserved across all AIEC isolates nor exclusive to this pathotype.

The prototype AIEC strain, LF82^6^, expresses the type 1 pili adhesin fimbriae adhesin H (FimH), which mediates adherence to ileal epithelial cells co-expressing carcinoembryonic antigen-related cell adhesion molecule 6 (CEACAM6)^28^. However, as CEACAM6 expression is comparable in both healthy and inflamed colonic tissues, additional host or microbial factors must underlie AIEC tropism^8,29^. This suggests that intestinal inflammation, dysbiosis, host–microbe interactions, diet, and epigenetic regulation collectively promote AIEC colonization and persistence in CD. Once internalized, AIEC can survive and replicate within macrophages without triggering host cell death^11^. This persistence involves a combination of bacterial virulence mechanisms, impaired intracellular killing, and defective autophagy linked to CD-associated polymorphisms in genes including nucleotide-binding oligomerization domain-containing protein 2 (*NOD2*), autophagy related 16-like 1 (*ATG16L1*), and protein tyrosine phosphatase non-receptor type 2 (*PTPN2*)^9,30^.

AIEC strongly contribute to chronic intestinal inflammation by activating innate immune pathways through microbial-associated molecular patterns such as flagellin and lipopolysaccharide^31^. Flagellin recognition by Toll-like receptor 5 (TLR5) and the NLR family CARD domain-containing protein 4 (NLRC4) inflammasome induces IL-1β and IL-6 secretion that perpetuates mucosal inflammation. Recent studies also implicate microRNA dysregulation in AIEC-driven inflammation: in *Il-10*-deficient mice infected with LF82, reduced expression of miRNA let-7b upregulated TLR4, enhancing secretion of IL-6, IL-8, and tumor necrosis factor-alpha (TNF-α) by colonic epithelial cells. Macrophages — abundant in inflamed CD tissue^32^ — also have a pivotal role in this process. During phagocytosis, AIEC LF82 triggers stress response and SOS genes that facilitate intracellular survival^33^. Infected macrophages secrete high levels of TNF-α, which paradoxically promotes further bacterial replication, creating a self-sustaining inflammatory loop^11^. Adaptive immune responses further reinforce this cycle: AIEC infection promotes T cell helper (T_H_)1/T_H_17 polarization, increasing interferon-gamma (IFN-γ) and IL-17A production that, in turn, enhance bacterial persistence^34^. Additionally, macrophages expressing fractalkine receptor (CX_3_CR1) facilitate bacterial translocation across the epithelium, amplifying mucosal immune activation^35,36^. Notably, AIEC infection of cultured IECs and macrophages induces exosome release that activates the NF-κB and mitogen-activated protein kinase pathways, augmenting inflammatory signaling^37^.

In the inflamed ileal mucosa of patients with CD, inflammation generates metabolites such as nitrate, tetrathionate, and lactate, whereas mucosal injury releases compounds including ethanolamine, fucose, and amino acids, which AIEC effectively exploits to enhance motility, adhesion, and virulence^9,29^. Exposure to bile salts induces transcriptional reprogramming and activates alternative metabolic pathways, such as the *eut* and *pdu* operons^38^. AIEC also exhibit enhanced biofilm formation, which is promoted by host-derived metabolites such as ethanolamine and linked to iron acquisition systems such as Ybt, contributing to bacterial persistence and antibiotic tolerance^39–41^. This metabolic adaptation confers a selective advantage in the nutrient-stressed inflamed gut, reinforcing a vicious cycle between metabolic reprogramming and chronic inflammation. Together, these findings highlight how AIEC persistence, immune modulation, and metabolic adaptation collectively sustain chronic intestinal inflammation and contribute to the development of inflammation-associated pathologies, including fibrosis and colitis-associated CRC.

## Colibactin: AIEC-derived genotoxin and its role in tumorigenesis

Persistent dysbiosis of the gut microbiome is increasingly recognized as a fundamental driver of tumorigenesis, shaping both the TME and host immune responses^42^. Microbial metabolites and small molecules influence multiple hallmarks of cancer — including genomic instability, chronic inflammation, and altered metabolism — integrating microbial activity into the broader framework of tumor biology^43^. Advances in sequencing and metagenomic analytics have deepened understanding of these host–microbe interactions, prompting the 2022 *Hallmarks of Cancer* update to include the microbiota as an emerging hallmark of tumor initiation and progression^44^.

The first identified bacterial carcinogen, *Helicobacter pylori*, illustrates how microbial infection can promote cancer. Its cytotoxin-associated gene A (CagA) virulence factor reprograms gastric epithelial cells toward proliferation and survival, thereby enhancing inflammation and gastric cancer risk^45^. Similar oncogenic mechanisms are now recognized among gut-resident bacteria linked to CRC. Over recent decades, evidence has shown that gut microbiota actively contribute to carcinogenesis by modulating the TME, epithelial remodeling, and mucosal immunity^42,46^. In CRC, specific species — including enterotoxigenic *B. fragilis*, *F. nucleatum*, and polyketide synthase (PKS)-positive (*pks*⁺) *E. coli* — have been causally associated with tumor initiation and progression^18,23,42,46,47^. *Pks*⁺ *E. coli* contribute to carcinogenesis through production of the genotoxin colibactin, which causes DNA damage and generates characteristic mutational signatures in CRC^24,48–50^. Notably, *pks*⁺ *E. coli* are enriched in inflamed and neoplastic mucosa — present in ~20% of healthy individuals, ~40% of patients with IBD, and ~60% of patients with CRC^23,51^.

### Colibactin genotoxicity

Colibactin is a secondary metabolite encoded by the *pks* genomic island, a 54-kb hybrid PKS–non-ribosomal peptide synthetase biosynthetic gene cluster comprising core enzymatic genes and accessory factors (*clbA–S*)^24^. The *pks* island is predominantly found in *E. coli* strains of phylogenetic group B2, which includes both commensal and pathogenic lineages such as AIEC. Despite its extreme chemical instability and the challenges associated with its isolation, biochemical and structural studies have elucidated its mode of action. Colibactin contains an electrophilic cyclopropane “warhead” that reacts with host DNA, causing alkylation of the N3 position of adenine and forming covalent colibactin–DNA adducts^52,53^. These adducts can bridge complementary DNA strands to generate interstrand crosslinks (ICLs) and, upon collapse of the replication fork, lead to the formation of DNA double-stranded breaks (DSBs)^24,54^. Such lesions obstruct DNA replication and activate canonical DNA damage response pathways in host epithelial cells.

Consistently, infection of epithelial cells with *pks*^+^ *E. coli* triggers ataxia-telangiectasia mutated (ATM) and ATM-related and Rad3-related DNA damage response signaling, marked by induction of γH2AX and G2-phase cell-cycle arrest^23,24,48^. Colibactin-induced DNA damage also recruits Fanconi anemia group D2 protein (FANCD2) to γH2AX foci, implicating the Fanconi anemia repair pathway in the resolution of ICLs^55^. Persistent or inadequately repaired lesions ultimately result in chromosomal aberrations, micronuclei formation, and sustained genomic instability^48,56^. Through these mechanisms, chronic exposure to colibactin-producing *E. coli* increases the mutational burden and transformative potential of colonic epithelial cells, establishing a direct molecular link between bacterial genotoxicity and tumor initiation.

Colibactin-induced DSBs occur preferentially at AT-rich hexameric sequence motifs and generate a distinct mutational pattern characterized by single-base substitution (SBS, T > N) and short insertion–deletion (ID) in AT-rich regions^49,50^. These patterns are found in mutational hot spots in *APC* and other CRC driver genes, linking colibactin exposure to oncogenic mutagenesis. Large-scale genomic analyses have confirmed that colibactin-associated mutational signatures — designated SBS88 and ID18 — are particularly enriched in early-onset CRC, suggesting that early-life exposure to *pks*⁺ bacteria may influence lifetime cancer risk^57^. In organoid models, transient infection with *pks*⁺ *E. coli* causes Wnt-independent proliferation, *TP53* mutations, and widespread chromosomal damage^56^. Although colibactin-induced lesions can be repaired, inadequate resolution leads to persistent genomic instability. Interestingly, these mutational signatures are also detected in healthy colonic epithelium^58^, and *pks*⁺ *E. coli* do colonize some normal microbiota, indicating that additional host or environmental factors determine progression from DNA damage to malignancy.

### *Pks⁺* AIEC and inflammation-driven CRC

Inflammation and dysbiosis create a permissive niche for *pks*⁺ AIEC expansion and mucosal colonization, thereby amplifying colibactin activity. In patients with IBD or CRC, *pks*⁺ AIEC are frequently isolated from colonic mucosa, in which the inflammatory milieu fosters a pro-neoplastic environment that promotes dysplasia and colitis-associated cancer. Colonization with *pks*⁺ AIEC enhances CRC development through colibactin activity, which was demonstrated in several mouse models^23,59–61^. The initial demonstration with the *pks*^+^ AIEC strain NC101 showed that mono-association of azoxymethane (AOM)-treated *Il10*⁻/⁻ mice induces invasive adenocarcinoma, whereas a *pks*-deficient mutant markedly reduces tumor burden and invasion despite equivalent inflammation, identifying colibactin-mediated DNA damage as the principal driver^23^. This carcinogenic effect requires inflammation via adaptive-immune context, as it disappears in un-inflamed *Il10*⁻/⁻;*Rag2*⁻/⁻ mice^61^. In colitis-associated cancer models, AOM/dextran sodium sulfate (DSS) and AOM/*Il10*⁻/⁻, *pks*⁺ NC101 accelerates tumorigenesis, whereas metabolic or microbiota-targeted interventions mitigate disease^60,62^. In *Apc*^Min/+^ and *Apc*^Min/+^;*Il10*⁻/⁻ models, colibactin-driven tumorigenesis correlated with inflammation and microbiota composition^59^.

Beyond direct genotoxicity, colibactin can modulate the tumor microenvironment. In AOM/DSS and xenograft CRC models, *pks*⁺ *E. coli* induced a senescence-associated secretory phenotype (SASP) characterized by altered miRNA expression and secretion of growth factors that stimulate proliferation of neighboring uninfected epithelial cells^63^. Chronic infection with the *pks*⁺ *E. coli* 11G5 strain in *Apc*^Min/+^ mice further promoted a pro-carcinogenic immune milieu by decreasing cytotoxic T cells and increasing regulatory T cells, resulting in tumor immune evasion and resistance to immunotherapy^64^. Collectively, these findings demonstrate that colibactin drives carcinogenesis through epithelial genotoxicity amplified by inflammation and microenvironmental cues. These dependencies highlight potential therapeutic levers — controlling inflammation, editing the microbiota, and directly targeting the colibactin pathway — to prevent or treat colibactin-driven CRC.

## AIEC-produced siderophores including Ybt

Fibrosis is a well-recognized pathological consequence of chronic intestinal inflammation in IBD. In CD, inflammation follows a relapsing–remitting course^65^. With increasing disease duration, the risk of fibrosis progressively rises, often independent of active inflammation. Approximately half of patients eventually develop fibrostenotic strictures that frequently necessitate endoscopic dilation or surgical resection. However, surgery does not cure the underlying fibrotic process, and postoperative recurrence of inflammation and stricture formation remains common, contributing to chronic morbidity and diminished quality of life^66^. Fibrosis is a multifactorial process characterized by excessive deposition of ECM — including overproduction of fibrillar collagens, increased fibronectin assembly, and lysyl oxidase (LOX)-mediated crosslinking — and scar formation driven primarily by activated stromal cells such as myofibroblasts. ECM accumulation reflects not only enhanced matrix synthesis but also reduced degradation owing to disrupted matrix metalloproteinase (MMP)–tissue inhibitor of MMP (TIMP) balance, leading to progressive architectural stiffening that reinforces fibroblast activation and perpetuates tissue remodeling^67^. Biologic therapies targeting TNF, integrins, and interleukins have markedly improved short-term inflammatory control in CD^66^. However, their long-term impact on the pathophysiological mechanisms that drive intestinal fibrosis remains poorly understood, and this knowledge gap continues to impede the development of effective treatments specifically targeting fibrotic remodeling. Emerging evidence suggests that the gut microbiota can promote fibrosis both directly — through activation of myofibroblasts — and indirectly — through persistent inflammation and immune–stromal crosstalk^14^. Advances in multi-omics technologies are now illuminating fibrosis-associated pathways, opening opportunities for mechanism-based therapeutic strategies.

### AIEC and fibrosis

Dysbiosis creates a permissive environment for AIEC, enabling its colonization and persistence within the inflamed intestine. AIEC expansion is strongly associated with exacerbation of intestinal inflammation and fibrotic remodeling, driven by sustained activation of immune and stromal pathways^12–16^. Consistent with this, colonization with diverse AIEC strains induces intestinal inflammation and prolonged AIEC infection leads to persistent inflammation and immunopathology throughout both the small and large intestines, characterized by T_H_1 and T_H_17 cytokine production and subsequent cecal and colonic fibrosis — recapitulating key features of human CD^13,68^. Comparative genomic analyses across diverse AIEC isolates from humans, dogs, and mice revealed enrichment of genes involved in propanediol utilization and iron acquisition^69^. These metabolic and virulence-associated traits enhance epithelial invasion and macrophage survival, amplifying inflammatory and fibrotic responses. Flagellin from AIEC LF82 upregulates the epithelial IL-33 receptor ST2, enabling IL-33-dependent fibrotic remodeling^16^. Engagement of IL-33 with ST2 activates NF-κB and mitogen-activated protein kinase signaling, driving pro-inflammatory cytokine production and TGF-β-mediated collagen deposition in the ECM. In parallel, AIEC use metabolic strategies that support survival in the nutrient-restricted and metal-restricted inflamed gut — processes particularly relevant to fibrosis pathogenesis.

### Metal homeostasis and siderophore systems

Transition metals are indispensable cofactors for numerous enzymatic and structural processes in both hosts and microbes^70–72^. Because metal availability regulates microbial growth and host defense, both hosts and pathogens have evolved competing strategies to control metal access. During infection, the host limits microbial access to essential metals through sequestration by high-affinity binding proteins such as calprotectin, lipocalin, lactoferrin, transferrin, and ferritin — a process termed nutritional immunity^70,73^. This defense restricts bacterial proliferation by depriving pathogens of iron, zinc, and other metals at the host–pathogen interface^72,74,75^. To counteract this, bacteria deploy specialized metal-acquisition systems, among which siderophores are the most efficient. Siderophores are low-molecular-weight chelators that bind ferric iron (Fe^3+^) with extremely high affinity and deliver it back into the bacterial cell via dedicated transporters^76,77^. Structural diversity — including catecholate, carboxylate, and hydroxamate types — enables bacteria to scavenge iron across various niches and contributes to colonization by both pathogenic and commensal *E. coli*^78^.

In IBD, AIEC depend heavily on siderophore-mediated iron acquisition to persist within the inflamed gut, where lipocalin and lactoferrin create an iron-restricted milieu. AIEC strains commonly produce siderophores such as enterobactin (Ent) and Ybt, which enhance epithelial invasion, intracellular survival in macrophages, and inflammatory potential.

### Yersiniabactin and fibrosis

Among siderophore systems, Ybt has a particularly important role under inflammatory conditions. Ybt is assembled through enzymatic machinery encoded on genes of the high-pathogenicity island (HPI) and is overexpressed in human, canine, and murine AIEC strains^69,79^. Its biosynthesis via HPI genes is similar to PKS–non-ribosomal peptide synthetase enzyme clusters that produce colibactin. Similar to other siderophores, Ybt captures iron; however, unlike the ubiquitous Enterobacteriaceae siderophore Ent, it is lipocalin-2 (Lcn2)-evasive, allowing Ybt⁺ pathogens to circumvent host nutritional immunity and gain a competitive advantage in the gut. In gnotobiotic *Il10*⁻/⁻ mice, Ybt-producing AIEC NC101 elicit inflammation-associated fibrosis^12^. These studies underscored a mechanistic link between Ybt-mediated metal acquisition and intestinal fibrosis, whereby bacterial production of Ybt was required for fibrosis, but bacterial utilization was not. These findings suggest that Ybt-metal sequestration directly targets the host. Beyond iron, Ybt can chelate other transition metals — including zinc, copper, and nickel^80–82^ — suggesting broader metallophore activity. Recently, we demonstrated that Ybt-producing AIEC NC101 sequesters zinc from macrophages, activating the transcription factor hypoxia-inducible factor 1-alpha (HIF-1α) via inhibition of its metal-dependent upstream regulator prolyl-hydroxylase 2 (PHD2)^15^. Enhanced macrophage HIF-1α signaling induces pro-fibrotic macrophages that drive fibroblast activation and pro-fibrotic gene expression, promoting intestinal fibrosis. Although microbiota-derived metabolites and metallophores are increasingly recognized as mediators of chronic inflammatory diseases, the precise mechanisms by which these molecules influence host–microbe interactions remain incompletely understood. Targeting siderophore systems such as Ybt may thus represent a promising strategy to modulate dysbiosis, inflammation, and intestinal fibrosis in CD.

## AIEC infection induces inflammation-associated chronic intestinal disease (CRC and fibrosis) through shared mechanisms

**Fig. 1:**
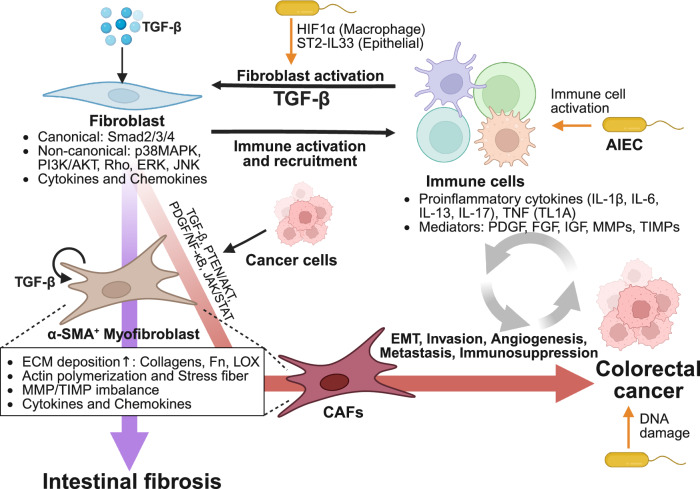
Crosstalk between fibroblasts and immune cells in intestinal fibrosis and colorectal cancer. Chronic intestinal inflammation creates a microenvironment in which fibroblasts engage in bidirectional crosstalk with infiltrating immune cells. Immune-derived pro-inflammatory and pro-fibrotic mediators — most notably TGF-β — activate fibroblasts through canonical Smad2/3/4 signaling and multiple non-canonical pathways. Activated fibroblasts recruit additional immune cells, forming a feed-forward loop that amplifies inflammation and drives differentiation into α-SMA⁺ myofibroblasts, leading to increased ECM deposition, stress-fiber formation, MMP/TIMP imbalance, and cytokine release. In parallel, cancer cells convert fibroblasts into CAFs via TGF-β-dependent and oncogenic pathways, enabling EMT invasion, angiogenesis, and immunosuppression. These fibroblast programs in fibrosis and tumorigenesis arise from shared stromal–immune mechanisms centered on TGF-β signaling. AIEC-derived signals can engage this overlapping pathway network, linking microbial dysbiosis to both intestinal fibrosis and colorectal cancer progression. AIEC adherent-invasive *Escherichia coli*, AKT protein kinase B, CAF cancer-associated fibroblast, ECM extracellular matrix, EMT epithelial–mesenchymal transition, ERK extracellular signal-regulated kinase, FGF fibroblast growth factor, Fn fibronectin, HIF1α hypoxia-inducible factor 1-alpha, IGF insulin-like growth factor, IL interleukin, JAK Janus kinase, JNK Jun N-terminal kinase, LOX lysyl oxidase, MAPK mitogen-activated protein kinase, MMP matrix metalloproteinase, NF-κB nuclear factor kappa-light-chain-enhancer of activated B cells, PDGF platelet-derived growth factor, PI3K phosphoinositide 3-kinase, PTEN phosphatase and tensin homolog, Rho Ras homolog family, Smad suppressor of mothers against decapentaplegic, STAT signal transducer and activator of transcription, ST2 suppression of tumorigenicity 2 (IL-33 receptor), TGF-β transforming growth factor-beta, TIMP tissue inhibitor of metalloproteinase, TL1A TNF-like ligand 1A, TNF tumor necrosis factor, α-SMA alpha-smooth muscle actin. Picture created with BioRender.com.

Chronic colonization by AIEC sustains a persistent inflammatory state that mechanistically links intestinal fibrosis and CRC. Continuous activation of innate and adaptive immunity drives repetitive cycles of cytokine release, barrier disruption, and epithelial–stromal injury, ultimately promoting both fibrostenotic remodeling and neoplastic transformation. These processes mirror the broader pathophysiology of CD, which features transmural inflammation, dysregulated wound healing, and progressive ECM accumulation underlying fibrostenotic strictures^66^. Single-cell analyses reveal that intestinal mesenchymal cells — which normally support epithelial maintenance and immune balance — undergo a profound shift during chronic inflammation^83^. Homeostatic subsets collapse, and pathogenic fibroblast populations emerge, characterized by expression of TNF superfamily member 14 (TNFSF14), IL-33, and LOX, contributing to impaired epithelial maturation, oxidative stress, and sustained inflammatory remodeling. Therefore, fibroblasts are now recognized as immune-interacting stromal cells rather than passive structural components — they sense microbial and inflammatory cues, secrete cytokines and chemokines, and orchestrate immune–stromal communication^84,85^. Across AIEC infection, CD, and colitis-associated CRC, a shared stromal–immune axis becomes apparent. Activated fibroblasts, ECM stiffening, and inflammation-driven oxidative and genotoxic stress collectively create a microenvironment that supports both fibrosis progression and tumor development, as illustrated in Fig. 1.

### Immune–fibroblast interactions, ECM remodeling, and TGF-β signaling in fibrosis and CRC

Within this chronic inflammatory niche, fibroblasts engage in extensive bidirectional crosstalk with infiltrating immune cells (Fig. 1). Epithelial cells, stromal cells, and immune cells secrete pro-fibrotic mediators, including TGF-β1; IL-1, IL-6, IL-17, IL-4, IL-13, IL-34; and members of the TNF family, which activate resident fibroblasts and drive their differentiation into ECM-secreting myofibroblasts that contribute to intestinal fibrosis^65,66^. Activated macrophages release pro-fibrotic mediators — including TGF-β and other growth factors — that expand myofibroblasts and drive excessive ECM deposition^66,86^. T cell-derived cytokines reinforce this cascade, enhancing macrophage–fibroblast crosstalk and boosting TGF-β1-dependent fibrogenic signaling^66^. In this inflammatory setting, AIEC exacerbate the fibrotic response by enhancing TGF-β signaling. AIEC induction of IL-33–ST2-dependent epithelial activation promotes IL-13 secretion of T_H_2 polarization. This IL-13-mediated amplification of TGF-β activity further drives fibroblast activation, ECM accumulation, and fibrosis^16^. TGF-β1 activates canonical signaling in fibroblasts, including suppressor of mothers against decapentaplegic (Smad) together with non-canonical stress kinases and cytoskeletal remodeling cascades that collectively promote myofibroblast differentiation and ECM production^66,87^. Mechanical tension generated by contracting myofibroblasts further releases latent TGF-β1, creating a self-perpetuating fibrotic loop^88^. Fibroblast-derived chemokines recruit immune cells to sustain inflammation, and additional cytokine signaling further reinforces fibroblast activation through a feed-forward inflammatory loop^84^.

The same TGF-β-dependent logic governs stromal remodeling in CRC. In normal and early neoplastic epithelium, canonical Smad signaling maintains cytostasis and genomic stability. As tumors progress, however, hypoxia, inflammatory cytokines, and ECM stiffening shift TGF-β activity toward non-canonical pathways^87,89^. These cascades increase integrin activation, contractility, and continuous ECM deposition, resulting in a progressively rigid tumor stroma. Elevated matrix crosslinking and accumulation of fibrillar components create a mechanically activated niche that promotes EMT, local invasion, and metastatic progression. Parallel immunologic outcomes emerge, as TGF-β-driven stromal remodeling in tumors favors immunosuppression, including expansion of regulatory and suppressive immune cells^89^, and resistance to chemotherapy, radiotherapy, and immunotherapy. Thus, the same pro-fibrotic machinery that sustains chronic intestinal fibrosis is repurposed during CRC progression to support malignant stromal remodeling and immune evasion. However, these mechanisms remain largely un-characterized in the intestine.

### Cancer-associated fibroblasts

Tumor cells engage stromal, immune, vascular, and ECM components to construct a complex TME^90^. Among these, cancer-associated fibroblasts (CAFs) have emerged as central orchestrators of ECM remodeling and tissue dynamics^91,92^. Malignant cells secrete pro-fibrotic and inflammatory mediators that convert resident fibroblasts into CAFs through the same pathways that also regulate EMT, underscoring the parallel plasticity of epithelial and stromal compartments during tumor progression (Fig. 1). Activated CAFs, in turn, secrete collagen, LOX, MMPs, and TIMPs, generating dense, stiff matrices that promote tumor cell proliferation, invasion, angiogenesis, and immune evasion. Tumor-derived cues further reinforce CAF activation, creating a feed-forward stromal loop in which malignant and fibroblastic compartments increasingly sustain tumor-promoting remodeling.

In CD, chronic inflammation similarly drives the emergence of CAF-like fibroblasts with increased contractility and pro-inflammatory secretory profiles, linking fibrotic remodeling to a tumor-permissive stroma. Recent single-cell analyses identified TWIST1⁺/fibroblast activation protein alpha⁺ (FAP⁺) fibroblast subsets in fibrostenotic CD lesions that share transcriptional and functional features with CAFs^93^. Given the pivotal role of TWIST1 in EMT and tumor progression^94^, these findings highlight stromal reprogramming as a shared pathogenic mechanism connecting fibrosis and tumorigenesis. Recent work demonstrates that *F. nucleatum* directly reprograms CRC-associated fibroblasts through a TLR2–YAP axis that upregulates connective tissue growth factor and remodels the tumor stroma, thereby promoting angiogenesis and tumor progression^95^. *F. nucleatum*-activated CAFs secrete pro-inflammatory cytokines such as CXCL1, IL-6, and IL-8, accompanied by elevated reactive oxygen species and heightened metabolic activity^96^. Although evidence for other gut microbes — including AIEC or *pks*⁺ *E. coli* — acting through CAF-centered mechanisms remains limited, several observations support this possibility. AIEC exacerbate intestinal fibrosis and upregulate epithelial vascular endothelial growth factor/HIF-1α programs^97^, suggesting microbe-driven angiogenesis within a remodeled matrix. Colibactin-producing *pks*⁺ *E. coli* induces DNA damage and SASP programs enriched in IL-6, IL-8, and TGF-β — cytokines known to activate or reinforce CAF-like phenotypes. Collectively, AIEC may function as upstream microbial triggers that prime intestinal fibroblasts toward CAF-like status, bridging fibrosis and tumorigenesis via matrix stiffening, aberrant angiogenesis, and inflammation-linked genomic instability. Consequently, targeting fibroblast activation and stromal remodeling represents a promising anti-fibrotic and potentially antitumor approach for patients with IBD, although further investigation into CAF biology and functional heterogeneity will be essential to optimize such strategies.

## Co-regulation of colibactin and Ybt in cancer progression

The biosynthetic pathways of colibactin and siderophores share a key enzymatic dependency on phosphopantetheinyl transferase (PPTase), the first enzyme required for their biosynthesis. *E. coli* encodes two such enzymes: EntD, a core-genome PPTase required for siderophore synthesis, and ClbA, encoded within the *pks* island. Martin et al.^98^ demonstrated that ClbA can substitute for EntD, activating both colibactin and siderophore biosynthetic enzymes. Strains lacking both *entD* and *clbA* are avirulent in infection models, whereas expression of either enzyme alone restores metabolite production and virulence. These findings establish a metabolic interdependence between the *pks* and HPI systems, suggesting that their genomic colocalization confers biochemical redundancy and coordinated regulation of secondary metabolism. This shared enzymatic axis provides a mechanistic basis for co-regulation: when *clbA* is induced — such as during host colonization or environmental stress — both genotoxic and metal-acquisition pathways can be co-activated. Therapeutically, inhibition of ClbA could therefore represent a dual-target strategy to suppress both colibactin and Ybt biosynthesis.

In phylogroup B2 *E. coli*, including AIEC, the colibactin and HPI-encoded Ybt loci are highly conserved, and more than 98% of *clb*-positive isolates also harbor Ybt biosynthesis genes^99^. The *pks* and HPI islands often integrate together on integrative and conjugative elements that mediate horizontal gene transfer among Enterobacteriaceae^99^, enabling their co-acquisition and stable maintenance. Dual-positive (*pks*⁺*/ybt*⁺) AIEC strains are enriched in patients with IBD, suggesting that the combination of colibactin-mediated genotoxicity and Ybt-driven metal acquisition provides a synergistic fitness advantage in the inflamed or nutrient-restricted intestinal environment. Under stress conditions, AIEC strains form robust biofilms that enhance intracellular persistence, with the HPI-encoded Ybt system required for optimal biofilm development and survival within host cells^9,40^. Tumor-associated mucosal biofilms enriched in *E. coli* have also been identified in familial adenomatous polyposis, linking biofilm-driven persistence and toxin production to colorectal tumorigenesis^100^. This biofilm-associated activation of Ybt connects metal capture to AIEC survival and chronic inflammation, reinforcing bacterial persistence and tumor-promoting activity in the IBD microenvironment.

Beyond genomic colocalization, a global regulatory axis coordinates expression of colibactin and Ybt. The RNA-binding protein CsrA, part of the BarA–UvrY two-component regulatory system, represses both *clb* and *ybt* expression at the post-transcriptional level^101^. Inactivation of CsrA or activation of the BarA–UvrY pathway — via small RNAs that antagonize CsrA — simultaneously upregulates production of both metabolites. This shared regulatory module integrates environmental cues such as carbon source availability, oxidative stress, and nutrient limitation, all characteristic of the inflamed intestinal niche. Functionally, the BarA–UvrY–CsrA circuit ensures that energy-intensive secondary metabolites are produced only when they confer maximal benefit — namely, during inflammation or host interaction — providing a molecular explanation for the frequent co-expression of colibactin and Ybt in IBD-associated isolates.

The co-expression of colibactin and Ybt may establish a pathogenic feed-forward loop within the intestinal microenvironment. Ybt-mediated metal sequestration perturbs host metal homeostasis and enhances oxidative stress, sensitizing epithelial and immune cells to colibactin-induced DNA damage. Colibactin then induces DNA ICLs and DSBs, driving mutagenesis, genomic instability, and SASP-associated inflammation. In parallel, Ybt promotes fibroblast activation and intestinal fibrosis through the zinc–HIF-1α pathway described earlier^15^. Moreover, we observed that elevated mucus concentration or zinc supplementation downregulates *clbR* and *clbB* expression in *pks*^+^ AIEC, suggesting that zinc negatively regulates colibactin biosynthesis (J. Jeyachandran et al., UNC undergraduate honors thesis, 2025). Together, this intertwined metabolic and genotoxic circuitry may accelerate the progression from chronic inflammation to fibrostenosis and malignancy in the IBD setting.

## Therapeutic targeting of AIEC-derived pathogenesis

**Fig. 2:**
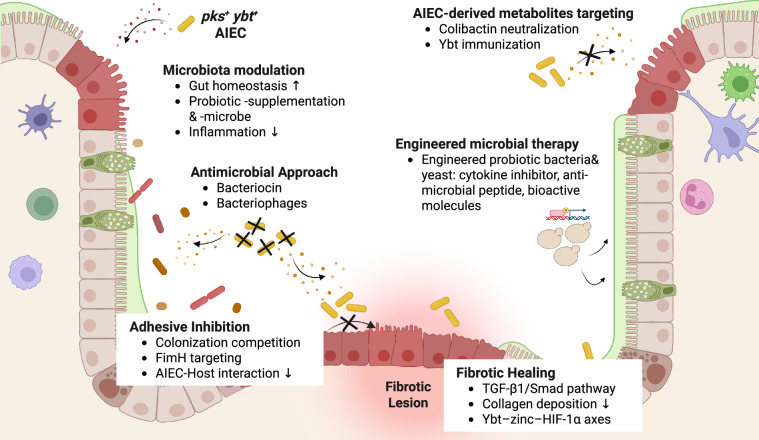
Therapeutic interventions targeting AIEC to reduce chronic inflammation-associated intestinal disease. Intestinal inflammation involves complex interactions among host cells and resident gut microbes, leading to inflammation and dysbiosis-sustaining changes to the intestinal microenvironment that promote fibrosis and colorectal cancer. AIEC are key drivers of these pathologies that may be targeted by multiple therapeutic approaches, mainly those that converge on AIEC metabolites colibactin and Ybt. Strategies include reducing collagen deposition and modulating TGF-β1/Smad or Ybt–zinc–HIF-1α pathways to promote fibrotic healing. Other approaches aim to eliminate carcinogenic *pks* ^+^ AIEC through antimicrobial agents or adhesive competition to minimize colonization. Novel engineered microbial therapies offer potential to target host and microbial factors to restore gut homeostasis and dampen shared signals driving inflammation, fibrosis, and colorectal cancer. AIEC adherent-invasive *Escherichia coli*, FimH fimbriae adhesin H, HIF-1α hypoxia-inducible factor 1-alpha, Smad suppressor of mothers against decapentaplegic, TGF-β transforming growth factor-beta, Ybt yersiniabactin*.* Picture created with BioRender.com.

Current therapeutic approaches for IBD and CRC encompass a broad range of pharmacologic and interventional strategies aimed at controlling inflammation, promoting mucosal healing, and preventing disease progression. In IBD, conventional agents such as aminosalicylates (for example, mesalamine), corticosteroids, and immunomodulators remain the therapeutic backbone^102^. Biologic therapies — including anti-TNF agents, anti-integrins, and anti-IL-12/23 antibodies — have markedly improved remission rates and mucosal healing^66^. More recently, oral small-molecule inhibitors that modulate key inflammatory signaling pathways have further expanded treatment options^103^. In CRC, standard management relies on surgical resection combined with fluoropyrimidine-based chemotherapy (5-FU or capecitabine) with oxaliplatin or irinotecan, which inhibits nucleic acid synthesis to suppress tumor proliferation. Additional targeted agents inhibit key regulators of cell-cycle progression, proteasome activity, and DNA repair^104^. Despite these advances, current therapies for both IBD and CRC remain constrained by variable patient responses, treatment resistance, systemic toxicity, and high costs. Importantly, these approaches primarily modulate host immune or tumor pathways but insufficiently address gut-specific factors — such as microbial dysbiosis, opportunistic pathogens such as AIEC, and inflammation-driven fibrosis — that critically link chronic intestinal inflammation to colorectal carcinogenesis. These unmet needs highlight the importance of microbiome-informed therapeutic design, as explored in the following sections (Fig. 2).

### Microbiome-targeted strategies to eliminate AIEC and disrupt AIEC–host interactions

Accumulating evidence identifies the gut microbiome as both a driver and a modulator of intestinal inflammation, underscoring its therapeutic potential. Strategies that restore microbial balance or directly interfere with AIEC–host interactions have gained particular attention for their capacity to reduce inflammation and limit pathogenic colonization.

#### Probiotics and microbiota modulation

Numerous animal and clinical studies have shown that probiotic supplementation can restore gut homeostasis and counteract dysbiosis in IBD and CRC. By enhancing beneficial microbes and suppressing pathogens such as AIEC, probiotics offer a promising strategy to mitigate AIEC-associated inflammation and colonization. Innovative biomaterial-based systems, such as mannose-functionalized microgels, demonstrate the feasibility of selectively capturing and clearing AIEC from the mucosa^105^. Moreover, enhancing the colonization capacity of probiotic microbes — which are notoriously poor colonizers in the presence of an established microbiota — may represent a way to enhance known probiotic-mediated protective mechanisms^106^. For example, beneficial commensals such as *Faecalibaculum rodentium* can protect against intestinal tumorigenesis by producing short-chain fatty acids such as butyrate, which can inhibit NF-κB signaling and suppress IL-6 and TNF expression, thereby limiting pro-tumorigenic inflammation^107,108^. Thus, maintaining a balanced microbiome with abundant beneficial strains that antagonize AIEC may reinforce mucosal homeostasis and disease resistance.

#### Inhibition of AIEC adhesion

Identifying patients who are AIEC-positive remains challenging owing to their genetic similarity to commensal *E. coli* and the absence of standardized diagnostic biomarkers, underscoring the need for improved detection methods^30^. Nevertheless, advances in understanding AIEC pathogenesis have enabled the development of therapeutic strategies that directly target bacterial adhesion and host interaction. *Lactobacillus* species reduce AIEC survival and adhesion within mucosal environments, whereas the yeast probiotic *Saccharomyces boulardii* prevents AIEC attachment to IECs through mannose–FimH interactions, thereby decreasing mucosal inflammation in experimental colitis models^109,110^. Small molecules targeting the AIEC adhesin FimH have been developed to block its binding to epithelial CEACAM6 receptors. Preclinical studies have developed small molecules that bind the carbohydrate-recognition site of FimH, thereby blocking its interaction with host glycans and reducing AIEC adhesion^111–113^. Among these, mannose-based glycopolymers and heptylmannoside derivatives show particularly high affinity for FimH. A clinical trial evaluating the FimH antagonist TAK-018 in patients with AIEC-positive CD was initiated but discontinued in 2023 owing to insufficient enrollment (NCT03943446). As *pks* ^+^ *E. coli* induce DNA damage only after attaching to epithelial cells through FimH, strategies that block AIEC adherence may offer not only anti-inflammatory benefits but also a potential cancer-preventive approach^114^. The continued development of FimH-targeted inhibitors represents a promising targeted approach to prevent AIEC colonization in susceptible individuals.

#### Antimicrobial and precision elimination approaches

Although antibiotics such as ciprofloxacin and rifaximin can transiently reduce AIEC, they also disrupt beneficial microbes and may worsen dysbiosis. More selective alternatives, including bacteriocins and bacteriophages, offer pathogen-specific control with minimal collateral effects. Colicins, bacteriocins produced by Gram-negative bacteria, eliminate competing strains through nuclease activity or cell wall disruption^115^. Colicins E1 and E9 exhibit potent activity against both extracellular and intracellular AIEC without host cytotoxicity^116^. Bacteriophages provide greater precision by selectively lysing target bacteria. A seven-phage lytic cocktail, EcoActive, eradicated 95% of 210 clinical AIEC isolates while preserving non-pathogenic *E. coli* in preclinical studies, illustrating the therapeutic promise of phage-based microbial control^117^.

#### Microbial metabolism and drug interaction

The gut microbiome has a crucial role in shaping how patients respond to therapy by directly influencing drug metabolism. For instance, certain commensal anaerobes can inactivate the IBD medication mesalamine through enzymatic acetylation, leading to the accumulation of inactive metabolites and reduced therapeutic efficacy in some patients^118^. Likewise, *E. coli*-derived β-glucuronidase can reactivate the chemotherapeutic drug irinotecan within the intestine, causing mucosal injury and severe diarrhea^119^. These examples highlight how microbial metabolism can both diminish drug efficacy and exacerbate toxicity. However, broad manipulation of the gut microbiota remains risky, as these microbes are essential for maintaining immune balance, epithelial integrity, and nutrient metabolism.

### Targeting AIEC-derived metabolites and fibrosis

Beyond microbiome modulation, targeting bacterial metabolites that directly promote inflammation, genotoxicity, and tissue remodeling has emerged as a promising therapeutic approach. Among AIEC-associated virulence factors, colibactin and Ybt are of particular interest, as they induce DNA damage, disrupt metal homeostasis, and contribute to chronic disease. Despite strong causal links to IBD, CRC, and fibrotic remodeling, no therapies currently exist that directly inhibit these pathways.

#### Colibactin inhibition

Certain anti-inflammatory therapies have been shown to indirectly suppress colibactin activity in preclinical models. Anti-TNF treatment reduces inflammation and tumor formation without altering *pks* ^+^ *E. coli* abundance, whereas mesalamine inhibits the microbial enzyme polyphosphate kinase, lowering colibactin production and DNA damage^62,120,121^. These observations suggest that host-directed anti-inflammatory drugs already approved for human use may attenuate microbial genotoxicity. The maturation of colibactin depends on the peptidase ClbP, which converts the inactive precolibactin into its genotoxic form^122^. In a murine AOM/DSS model colonized with *pks*^+^*E. coli*, administration of a boron-based ClbP inhibitor reduced DNA damage and tumor burden, supporting the feasibility of pharmacologic suppression of colibactin biosynthesis^123^. One of the key challenges in early studies was the expression and isolation of full-length, functional ClbP, a membrane-bound and intrinsically unstable enzyme^124^. It was not until recently that full-length ClbP was fully characterized in vitro^125^, leading to the development of boronic acid-based inhibitors that block catalytic activity in *E. coli* NC101 and prevent host cell-cycle arrest and DNA adduct formation^126^. Complementing enzyme-targeted strategies, engineered bacteria expressing the colibactin resistance protein ClbS on their surface can neutralize colibactin in the gut and attenuate *pks*^+^ *E. coli*-driven tumorigenesis^127^. These findings provide proof of concept that colibactin can be pharmacologically silenced to prevent DNA damage and tumorigenesis.

#### Yersiniabactin targeting

Parallel efforts have focused on siderophore biosynthetic enzymes as therapeutic targets. High-resolution crystal structures of a Ybt-associated cyclization domain have revealed the substrate-binding pocket and catalytic geometry, enabling structure-guided inhibitor design^128^. Ybt also exhibits strong affinity for transition metals such as copper. Using copper-64-labeled Ybt, researchers successfully imaged bacteria expressing the Ybt transporter, establishing a novel optical approach for tracking *E. coli* colonization in vivo^129^. Although not originally developed for therapy, this imaging strategy demonstrates how microbial metabolites can be leveraged for diagnostic or targeted-delivery applications through their intrinsic metal-binding properties. Traditional immunization approaches have also been used in preclinical models to target Ybt-producing bladder *E. coli* to treat urinary tract infection^130–132^. Immunization against the siderophore enterobactin reduced infection with the enteric pathogen *Salmonella*, as well as AIEC colonization and intestinal inflammation in mouse models^133,134^. Although further studies are needed, these approaches may offer new possibilities for treating human chronic intestinal disease.

#### Engineered microbial therapeutics

Synthetic biology has enabled the engineering of probiotic bacteria and yeast to deliver therapeutic molecules directly within the GI tract. Engineered organisms can secrete cytokine inhibitors, antimicrobial peptides, or bioactive molecules that modulate local inflammation and restore mucosal homeostasis. For example, *S. boulardii* expressing anti-TNFα nanobodies reduced tumor burden in AOM/*Il10*⁻/⁻ mice, demonstrating the potential of localized cytokine neutralization^135^. Engineered bacteria secreting pore-forming toxins such as cytolysin A or listeriolysin O induce apoptosis in tumor cells across multiple cancer models^136,137^, whereas biosensor microbes based on *Acinetobacter baylyi* have been designed to detect tumor-derived DNA using CRISPR-based discrimination^138^. In the context of IBD, *E. coli* Nissle 1917 has been engineered to express recombinant colicins targeting AIEC, providing a precision approach to selectively eliminate pathogenic strains^139^. The same chassis strain has also been modified to promote epithelial repair by secreting trefoil factor-based matrices, reducing inflammation and pro-inflammatory cytokine levels in preclinical colitis models^140^. Extending these principles, engineered microbes could locally deliver antifibrotic agents — such as naringin, pitavastatin, elafin, or TGF-β modulators — to attenuate tissue remodeling while minimizing systemic toxicity. Together, these advances illustrate how microbial engineering integrates targeted delivery, immune modulation, and biosensing to create a flexible platform for precision therapeutics. Such approaches hold strong potential for eliminating AIEC, reducing fibrosis and CRC risk, and restoring gut homeostasis.

#### Paired targeting of host and microbe in fibrosis

Strategies described earlier to target gut microbes and their metabolites could be paired with host-directed therapeutics. However, efforts to treat intestinal fibrosis remain limited. Most anti-fibrotic therapies target hepatic or pulmonary systems, with few addressing the gut^141^. Nonetheless, modulation of the TGF-β1/Smad pathway has shown promise in experimental colitis: TGF-β1 vaccination, pirfenidone, and Smad7-targeting oligonucleotides reduced collagen deposition and fibrotic remodeling in murine models^142–144^. However, as this pathway also governs epithelial restitution and wound healing, its inhibition may impair mucosal repair and, through immunosuppression, exacerbate inflammation and increase the risk of tumorigenesis^145^. Thus, therapeutic targeting of TGF-β signaling must carefully balance anti-fibrotic efficacy with the preservation of epithelial integrity and immune surveillance. The gut’s continuous exposure to microbial and dietary factors further complicates this balance, underscoring the need for gut-specific and context-dependent antifibrotic strategies. Building on these challenges, the mechanism we identified wherein AIEC-produced Ybt sequesters zinc from the host, stabilizing HIF-1α and sustaining pro-fibrotic cell signaling, suggests new therapeutic possibilities^15^. Targeting the Ybt–zinc–HIF-1α axis, through modulation of hydroxylase activity, restoration of zinc availability, and/or selective regulation of HIF-1α signaling, may represent a novel antifibrotic strategy in IBD.

## Conclusions

Chronic intestinal inflammation drives both fibrosis and tumorigenesis, and accumulating evidence indicates that the gut microbiota can critically influence this trajectory. Among inflammation-associated microbes, AIEC are strongly linked to CD. Strong evidences across many preclinical models indicate AIEC contribute to both intestinal fibrosis and colorectal carcinogenesis. AIEC-derived metabolites — particularly colibactin and Ybt — modulate epithelial, immune, and stromal responses, although the precise pathways through which they act have only recently begun to be resolved. A unifying link, however, is that chronic inflammation, fibrosis, and tumorigenesis converge on shared TGF-β-dependent stromal pathways and on fibroblast reprogramming into CAF-like phenotypes that promote malignant progression. Consequently, dual *pks*^+^*/ybt*^+^ AIEC strains may promote not only intestinal fibrosis but also CAF-mediated tumorigenesis through metabolite-driven remodeling of the microenvironment. However, the combined physiological impact of colibactin and Ybt — and their context-dependent effects — requires further investigation. Defining these mechanisms will be crucial for developing microbiota-informed therapies capable of targeting intestinal fibrosis as well as inflammation-associated CRC. Such therapies will offer new opportunities for treating fibrostenotic and CAF-enriched tumor states in patients with IBD.
